# Importance of Multimodal Testing for Novel ALK Fusions: A Case of *MTHFD1L‐ALK* Positive Lung Squamous Cell Carcinoma With Negative IHC

**DOI:** 10.1111/1759-7714.70348

**Published:** 2026-07-19

**Authors:** Huiwen Yang, Shuqiao Zheng, Jiaohui Pang, Jinming Yu, Linlin Wang

**Affiliations:** ^1^ Shandong First Medical University, Shandong Academy of Medical Sciences Jinan Shandong China; ^2^ Department of Radiation Oncology, Shandong Cancer Hospital and Institute Shandong First Medical University, Shandong Academy of Medical Sciences Jinan China; ^3^ Geneseeq Research Institute Nanjing Geneseeq Technology Inc. Nanjing Jiangsu China

**Keywords:** ALK inhibitor, ALK rearrangement, ALK‐MTHFD1L, next‐generation sequencing, non‐small‐cell lung cancer

## Abstract

Anaplastic lymphoma kinase (ALK) rearrangements are rare in lung squamous cell carcinoma (LSCC), with the clinical efficacy of ALK tyrosine kinase inhibitors (TKIs) in patients harboring uncommon ALK fusions remaining poorly characterized. We report here the first identification of a novel *MTHFD1L‐ALK* fusion in a 63‐year‐old male with a heavy smoking history diagnosed with stage IIIC LSCC. The patient initially received induction therapy with the ALK TKI iruplinalkib, achieving a best response of stable disease (SD). Although subsequent chemoradiotherapy yielded a partial response (PR), disease progression occurred after four cycles of maintenance iruplinalkib, with a progression‐free survival (PFS) of 8.28 months. Subsequent lorlatinib provided limited benefit, with disease progression at 5.3 months following treatment self‐discontinuation. Retrospective immunohistochemical staining of ALK (D5F3) was negative despite the positive genomic finding. This case demonstrates limited clinical benefit from ALK inhibitors in LSCC with this novel fusion, expands the known mutational spectrum in non‐small cell lung cancer (NSCLC), and underscores the critical importance of confirming novel fusions at the protein expression level through multimodal testing.

## Graphical abstract

We report the first case of a novel *MTHFD1L–ALK* fusion detected by DNA‐based NGS in a patient with stage IIIC lung squamous cell carcinoma who showed limited clinical benefit from ALK inhibitors. Retrospective ALK IHC (D5F3) was negative. This discordance between DNA‐based NGS and ALK IHC highlights the importance of multimodal confirmation of novel fusions before initiating targeted therapy.

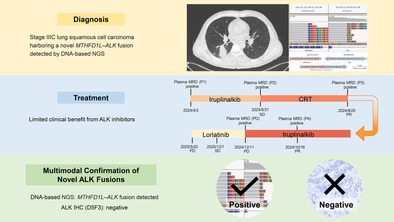

## 1 Introduction

Anaplastic lymphoma kinase (ALK) rearrangements occur in 3%–7% of non‐small cell lung cancers (NSCLC), predominantly adenocarcinoma. ALK‐positive lung squamous cell carcinoma (LSCC) represents an exceptionally rare molecular subset, accounting for 0%–2.5% of cases [1, 2, 3]. Emerging evidence suggests that the specific fusion partner critically influences clinical outcome, with over 90 distinct ALK fusion variants identified and the most prevalent partner *EML4* [4, 5]. Despite the remarkable efficacy of first‐ to third‐generation ALK tyrosine kinase inhibitors (TKIs) in ALK‐driven NSCLC, the data on ALK rearrangement in LSCC patients are limited and the therapeutic efficacy remains undefined in those individuals. Herein, we report a novel *MTHFD1L‐ALK* fusion identified in a patient with locally advanced LSCC, which expands the molecular spectrum of ALK rearrangements. We present the following case in accordance with the CARE reporting checklist.

## 2 Case Presentation

**FIGURE 1:**
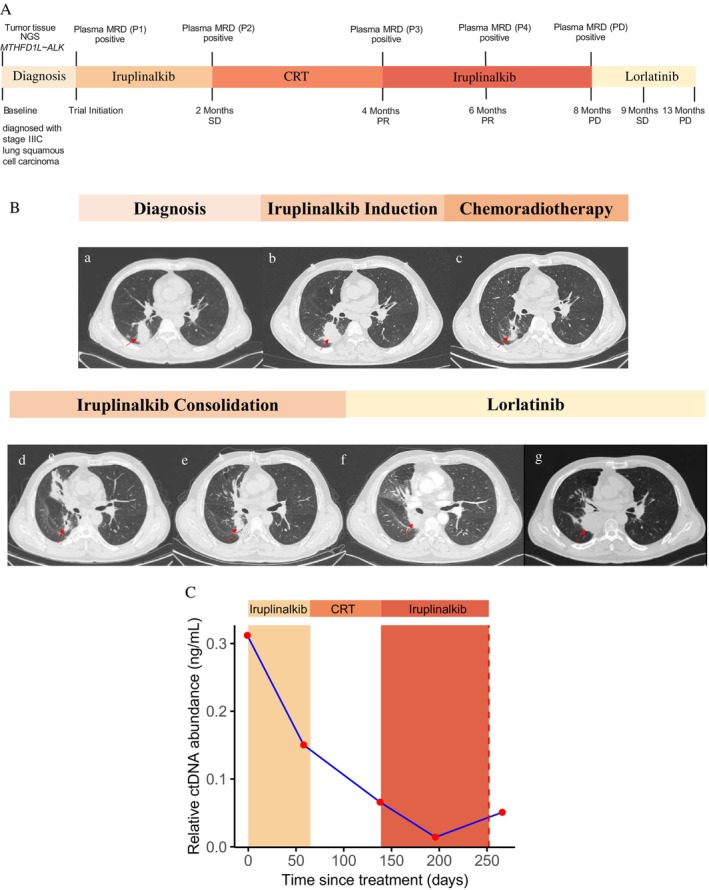
The treatment timeline and dynamic monitoring of chest computed tomography (CT) scans and plasma circulating tumor DNA (ctDNA). (A) The treatment timeline of the patient. (B) Images of chest computed tomography (CT) scans of the patient during the course of treatment. (a) At initial diagnosis (b) After 2 cycles of iruplinalkib induction (c) After 2 cycles of chemotherapy combined with radiotherapy (d) During iruplinalkib consolidation (e) Progressive disease after first‐line treatment (f) After 1 cycle of lorlatinib (g) Progressive disease after second‐line treatment. (C) Dynamic changes in ctDNA to monitor the clinical response.

A 63‐year‐old male with a 50‐year smoking history presented with a chronic cough. Both chest computed tomography (CT) and positron emission tomography‐computed tomography (PET‐CT) demonstrated a 3.0 × 5.0 cm mass in the lower lobe of the right lung, with right hilar and mediastinal lymph nodes and pleural metastases, without bone, liver, and brain metastases (Figure 1A,B‐(a)). The patient received a CT‐guided percutaneous lung biopsy, and pathological examination showed lung squamous cell carcinoma positive for P40, CK5/6, and negative for TTF‐1. Following multidisciplinary evaluation, the patient was staged as IIIC (T3N3M0) LSCC. Next‐generation sequencing (NGS) was performed on the biopsy specimen using GeneseeqPrime (Nanjing Geneseeq Technology Inc., China), a hybrid capture‐based assay covering all exons and selected introns of 425 cancer‐related genes (including *ALK* introns for fusion detection). The library was sequenced on the DNBSEQ‐T7 platform. The mean sequencing depth was 2271× with a limit of detection (LOD) of 1%. Mutations and fusions were manually validated using the Integrative Genomics Viewer (IGV).

The analysis identified a previously unreported *MTHFD1L‐ALK* fusion (M21:A12), and the specimen was positive for *TP53* exon 6 mutation. Based on the ALK‐positive status, this patient was enrolled in the phase II study of WX‐0593 combined with concurrent chemoradiotherapy in the treatment of unresectable locally advanced NSCLC with mutations of *ALK* or *ROS1*. This study was registered with https://ClinicalTrials.gov (identifier NCT05351320) on April 28, 2022. He received 2 cycles of oral iruplinalkib monotherapy (180 mg once daily with a 7‐day lead‐in phase at 60 mg once daily), and SD was achieved after this induction treatment (Figure 1B‐(b)). Subsequently, thoracic radiation therapy (56Gy/28f) was administered with concurrent 2 cycles of chemotherapy (paclitaxel and cisplatin), and the patient had a partial response in all lesions (Figure 1B‐(c)). After 4 cycles of iruplinalkib monotherapy, monitoring of molecular residual disease (MRD) demonstrated a rise in plasma circulating tumor DNA (ctDNA) level (Figure 1C), and the CT scan demonstrated significant disease progression with enlarged lymph nodes and increased size of the lung masses (Figure 1B‐(e)). The ctDNA analysis confirmed the persistence of the *MTHFD1L‐ALK* fusion, but no secondary *ALK* resistance mutations were detected. A tissue re‐biopsy was not performed due to patient refusal. The patient was withdrawn from the clinical trial. Subsequently, he switched to the third‐generation ALK inhibitor lorlatinib (100 mg once daily), and follow‐up scan showed he was in a stable condition after 1 cycle of lorlatinib treatment (Figure 1B‐(f)). However, the patient self‐discontinued the treatment after finishing two cycles and opted for traditional Chinese medicine. The subsequent CT scan at 5.3 months showed disease progression with a notable enlargement of the lung mass (Figure 1B‐(g)).

**FIGURE 2:**
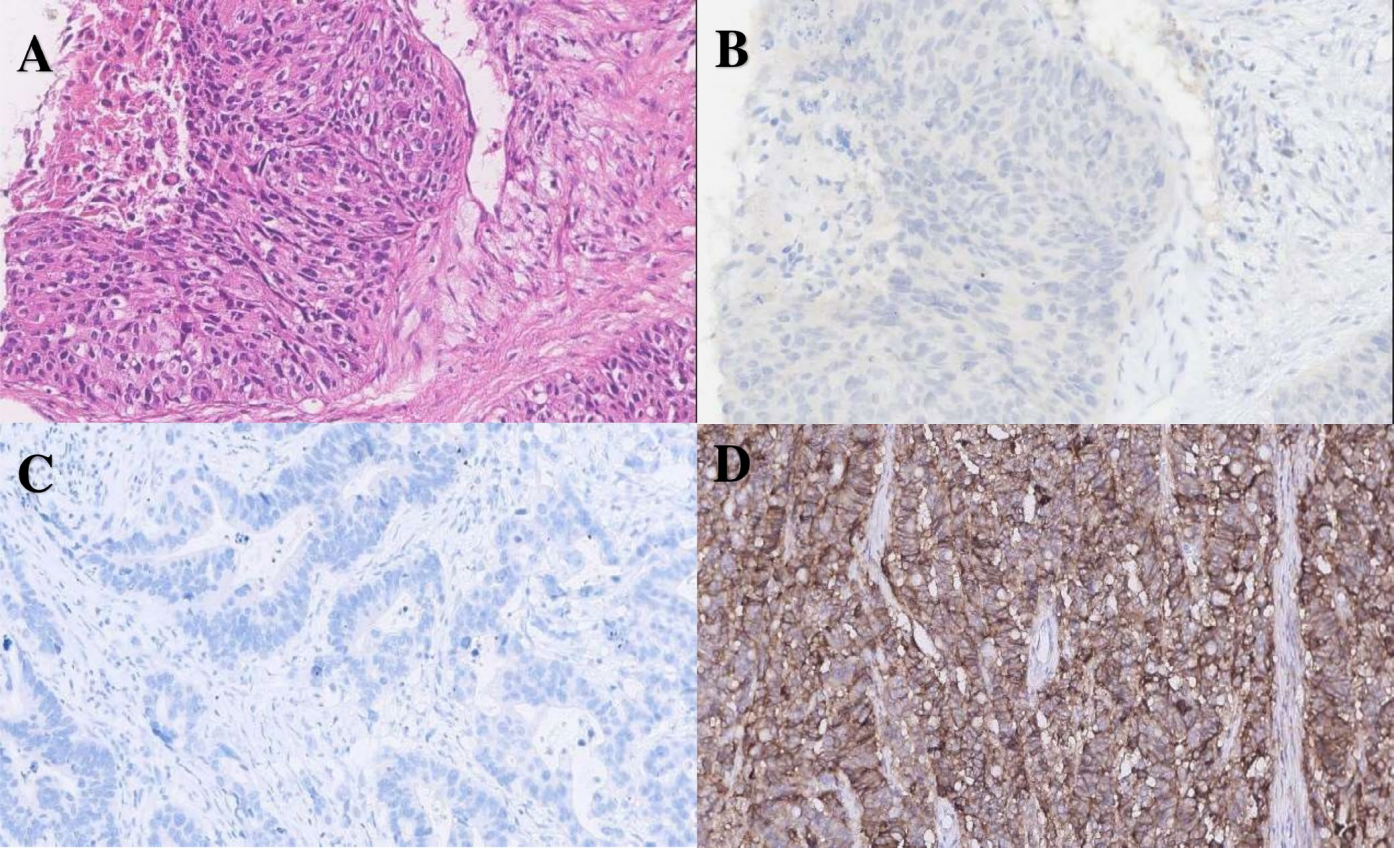
Pathology findings. (A) Hematoxylin and eosin‐stained biopsy specimen (H&E, 200×); (B) Immunohistochemical staining negative for ALK (D5F3) (200×); (C) Negative control for ALK (D5F3) (200×) (D) Positive control for ALK (D5F3) (200×).

Considering his disease progression, we retrospectively performed immunohistochemical staining of ALK (D5F3) on the tumor biopsy tissue, and it showed negative ALK expression (Figure 2).

## 3 Discussion

We report a novel *MTHFD1L‐ALK* fusion from a 63‐year‐old male with locally advanced lung squamous cell carcinoma. Iruplinalkib is a next‐generation ALK TKI approved by the National Medical Products Administration (NMPA) for ALK‐positive NSCLC [6, 7]. In the INNOVATION trial, iruplinalkib consolidation after definitive chemoradiotherapy achieved an objective response rate (ORR) of 86% and a 12‐month progression‐free survival rate of 100% during a median 17.7‐month follow‐up in part 1 of this study. However, this patient achieved only a PFS of 8.28 months with iruplinalkib‐based chemoradiotherapy and failed to respond durably to lorlatinib. This discordant outcome prompted us to investigate the structural and functional limitations of this specific fusion.

**FIGURE 3:**
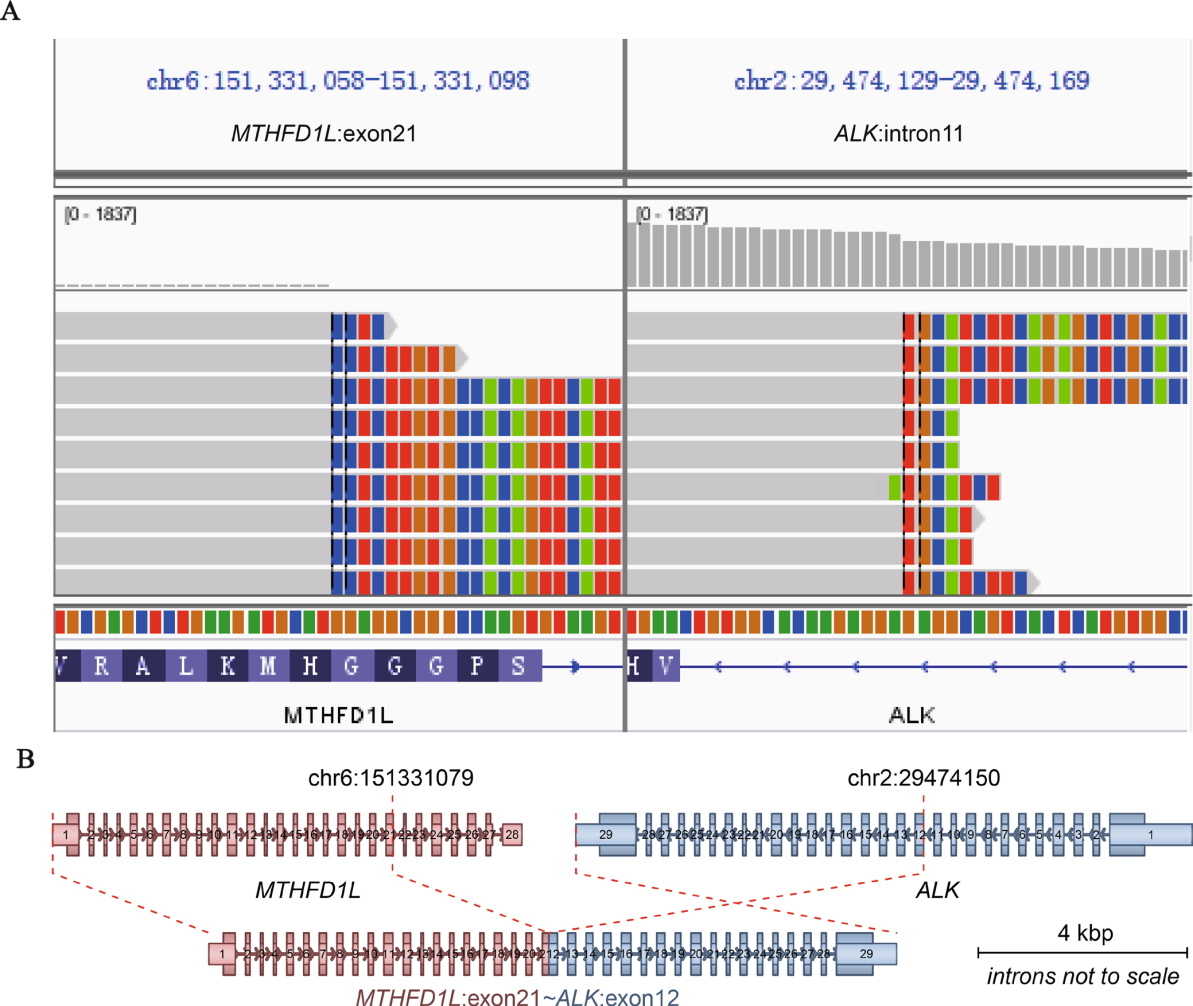
Molecular characterization of a novel *MTHFD1L‐ALK* fusion in lung squamous cell carcinoma (LSCC). (A) Next‐generation sequencing reads spanning the *MTHFD1L‐ALK* fusion breakpoint visualized using the Integrative Genomics Viewer (IGV). (B) Schematic representation of the fusion architecture showing exons 1–21 of *MTHFD1L* (chromosome 6, depicted in red) joined to exons 12–29 of *ALK* (chromosome 2, depicted in blue).

Given the limited clinical benefit, we performed retrospective analysis to verify the functional status of this fusion. The fusion in this case represents a novel translocation between exons 1–21 of *MTHFD1L* and exons 12–29 of *ALK*. Bioinformatic analysis of the breakpoint reads confirmed that this fusion is in‐frame and incorporates the complete intracellular kinase domain of ALK (Figure 3). While RNA sequencing would have been optimal to confirm transcript production, it could not be performed due to insufficient quantity. Therefore, we retrospectively performed IHC (D5F3), which revealed negative ALK protein expression despite the positive genomic finding. Several mechanisms may explain this genomic‐protein discordance, including transcriptional silence of the *MTHFD1L* promoter, translational failure, or post‐translational degradation of the unstable fusion product. We hypothesize that the latter is most likely, as MTHFD1L lacks the coiled‐coil domains found in canonical partners such as EML4 that are essential for stable dimerization [8]. This structural deficiency likely leads to rapid protein degradation and weak kinase activation, explaining both the IHC negativity and limited TKI efficacy.

Our findings align with emerging evidence that TKI sensitivity varies significantly depending on the fusion partner [9]. While canonical *EML4‐ALK*, *KIF5B‐ALK*, and *HIP1‐ALK* are typically sensitive to ALK inhibitors due to strong dimerization domains, variants such as *STRN‐ALK* and certain non‐reciprocal fusions have been associated with poorer outcomes or primary resistance [8, 10]. Our case adds to this body of evidence suggesting that *MTHFD1L‐ALK*, lacking key oligomerization motifs, may confer a weak driver phenotype with limited sensitivity to ALK inhibitors. Considering the limited efficacy of ALK TKIs and the PR achieved during chemoradiotherapy, the discordance between positive DNA‐NGS result and negative ALK protein expression by IHC suggested a potentially non‐functional fusion variant. Therefore, this case demonstrates that DNA‐level detection alone for novel fusion partners may be insufficient to reliably identify patients who will benefit from targeted therapy. For rare or novel fusion partners or atypical histologies such as LSCC, we advocate for the incorporation of complementary diagnostic modalities such as IHC or RNA‐NGS to confirm functional ALK fusion expression before initiating targeted therapy.

In this case, longitudinal ctDNA monitoring demonstrated persistently positive MRD levels that paralleled the clinical progression (Figure 1B,C), further confirming the lack of molecular clearance and the limited durability of the TKI response in this IHC‐negative variant.

## 4 Conclusions

In conclusion, this is the first report of a LSCC patient harboring an *MTHFD1L‐ALK* rearrangement, expanding the known mutational spectrum in NSCLC. Discordant genomic and immunohistochemical results highlight the necessity of multimodal testing to validate the protein‐level expression in rare molecular subsets.

## Author Contributions

**Jiaohui Pang:** data curation. **Shuqiao Zheng:** data curation. **Linlin Wang:** writing – review and editing. **Jinming Yu:** writing – review and editing. **Huiwen Yang:** writing – original draft, data curation.

## Funding

This study was supported by National Key Technologies Research and Development Program (Grant number 2022YFC2404605), National Natural Science Foundation of China (Grant number 82172865), Post‐Marketing Clinical Research Special Project on Innovative Drugs (Grant number WKZX2023Cx020012), Natural Science Foundation of Shandong Province (Grant number ZR2023LZL002, ZR2024MH007, and ZR2021LZL009), Collaborative Academic Innovation Project of Shandong Cancer Hospital (Grant number ZF002), and Noncommunicable Chronic Diseases‐National Science and Technology Major Project (Grant number 2024ZD0519904).

## Ethics Statement

The patient provided written informed consent for participation in the INNOVATION study and publication of anonymized clinical information and images, which was approved by the ethics committee of Shandong Cancer Hospital and Institute.

## Conflicts of Interest

Jiaohui Pang is an employee of Nanjing Geneseeq Technology Inc. All other authors declare that they have no conflicts of interest.

## Data Availability

The data that support the findings of this study are available from the corresponding author upon reasonable request.
